# A 17q24.3 duplication identified in a large Chinese family with brachydactyly‐anonychia

**DOI:** 10.1002/mgg3.1392

**Published:** 2020-06-25

**Authors:** Mohan Liu, Xueguang Zhang, Hongqian Liu, Ying Shen

**Affiliations:** ^1^ Department of Obstetrics/Gynecology Joint Laboratory of Reproductive Medicine (SCU‐CUHK) Key Laboratory of Obstetric Gynecologic and Pediatric Diseases and Birth Defects of Ministry of Education West China Second University Hospital Sichuan University Chengdu China; ^2^ State Key Laboratory of Biotherapy and Cancer Center West China Hospital Sichuan University and Collaborative Innovation Center Chengdu China; ^3^ Department of Obstetrics and Gynecology Key Laboratory of Obstetric Gynecologic and Pediatric Diseases and Birth Defects of Ministry of Education West China Second University Hospital Sichuan University Chengdu China

**Keywords:** 17q24.3 duplication, brachydactyly, brachydactyly‐anonychia, *KCNJ2*, *SOX9*

## Abstract

**Background:**

Brachydactyly (BD) is a rare autosomal dominant inherited disease characterized by shortness of the fingers and/or toes, which has been classified into the subtypes A–E. However, the exact cause and mechanism of BD remain to be illuminated. Here, we aim to reveal the clinical and genetic characteristics of a subtype of BD, brachydactyly‐anonychia.

**Methods:**

In this study, a large Chinese family with three members affected by brachydactyly‐anonychia was investigated. Both whole‐exome sequencing and microarray‐based comparative genomic hybridization (CGH) were performed on this family and the results of copy number variation (CNV) were verified by quantitative real‐time PCR (qPCR).

**Results:**

All the affected individuals showed short fingers and toes as well as missing nails; and the absence of middle phalanges in figure II‐V of the upper and lower extremities was observed by X‐ray examination. A duplication involving in the region of 17q24.3 was detected by CGH. The results of qPCR also represented this duplication in 17q24.3 in all the patients.

**Conclusion:**

In summary, our findings suggest that 17q24.3 duplication is the genetic cause of brachydactyly‐anonychia in this family, which support the prior report that brachydactyly‐anonychia is associated with 17q24.3 duplication, and further indicates the pathogenic correlation between BD and CNVs.

## Graphical abstract

We firstly identified a new subtype of brachydactyly in a large Chinese family carrying the similar duplication involving in regulatory region of SOX9 (17q24.3), which is important for the future gene diagnosis.

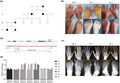

## 1 INTRODUCTION

Brachydactyly (BD) is a rare genetic disease with short fingers (or toes) due to metacarpal (and/or phalangeal) developmental abnormalities, usually involved in autosomal dominant inheritance pattern. According to the characteristics of finger deformity, it can be divided into A‐E types and different subtypes (Fitch, 1979). BD can occur alone or as part of complex malformation syndromes, which is often accompanied by other hand/foot deformities such as syndactyly/toe, polydactyly/toe, deficit deformities, and finger/phalangeal joint fusion (Mundlos S, 2009). Now, the pathogenic genes of BD type A, type B, and type C have been identified, and the related gene mutations have also been found in type D and type E (Burgess, 2001; Gong et al., 1999; Johnson et al., 2003; Oldridge et al., 1999, 2000; Schwabe et al., 2000). However, there are still some kind of BD with unclear etiology because of the rare reports.

In 2009, Kurth et al. (2009) reported seven affected individuals representing BD as well as absent nails in all digits, and suggested that the duplications of noncoding elements 5′ of *SOX9* (OMIM *608160) might be the cause of brachydactyly‐anonychia in the four families. Since then, no other studies have reported this disease any more. Intriguingly, here we identified a similar duplicated region in 17q24.3 involving putative regulatory elements of *SOX9* and the coding sequences of *KCNJ2* (OMIM *600681) and *KCNJ16* (OMIM *605722) in three Chinese patients with the same phenotype from a large family. Our findings provide further evidence of the pathogenicity of 17q24.3 duplications in brachydactyly‐anonychia.

## 2 MATERIALS AND METHODS

### 2.1 Patients

The proband is a female from an nonconsanguineous Chinese family. The family members of the patient were additionally recruited. All affected individuals in this family underwent the physical examination as well as the radiographic examination. The study was approved by the Ethical Review Board of West China Second University Hospital, Sichuan University. Informed consent was obtained from each subject in our study.

### 2.2 Whole‐exome sequencing

Using IDT The xGen Exome Research Panel v1.0 full exon capture chip and Illumina NovaSeq 6000 series sequencer, the whole exome of the proband was sequenced with high throughput, and the target sequence coverage was not less than 99%. Variants were removed if the following conditions were met: (a) the minor allele frequency is greater than or equal to 1% in ExAC Browser, gnomAD, or the 1,000 genome Project; (b) the variant is not predicted deleterious by SIFT, PolyPhen‐2, and MutationTaster tools; (c) the variant in noncoding exons, 3′ or 5′ untranslated regions, or intronic sequences except canonical splice sites.

### 2.3 Microarray‐based comparative genomic hybridization

The gDNA of the patients was segmented, and the sequencing library was constructed. The high‐throughput sequencing was carried out on the platform of Illumina HiSeq 2000, and the resolution of copy number variation (CNV) was 0.1 Mb. After comparing the sequenced fragments with the human reference genome (hg19), the duplications or deletions of patients were screened (excluding high repetition and pyknosis acrocentric and low proportion chimerism), and the detected data were matched with the database of genomic variants (DGV), the database of chromosomal imbalance and phenotype in humans using ensemble resources (DECIPHER) and the international standard cytogenomic array (ISCA). The online Mendelian Inheritance in Man (OMIM) and PubMed database were consulted, and the significance of patient CNV was obtained.

### 2.4 Quantitative real‐time PCR amplification

Peripheral blood of all the family members and the normal controls were collected, and gDNA was extracted by blood genome extraction kit (QIAGEN). Its concentration and purity were determined by NanoDrop2000 (Thermo Company). Primers were designed within and outside the 17q24.3 duplication region, and the relative copy number in gDNA of patients and normal controls was detected by quantitative real‐time PCR (qPCR). The information of the primers is given in Table S1.

## 3 RESULTS

### 3.1 Clinical report

**Figure 1:**
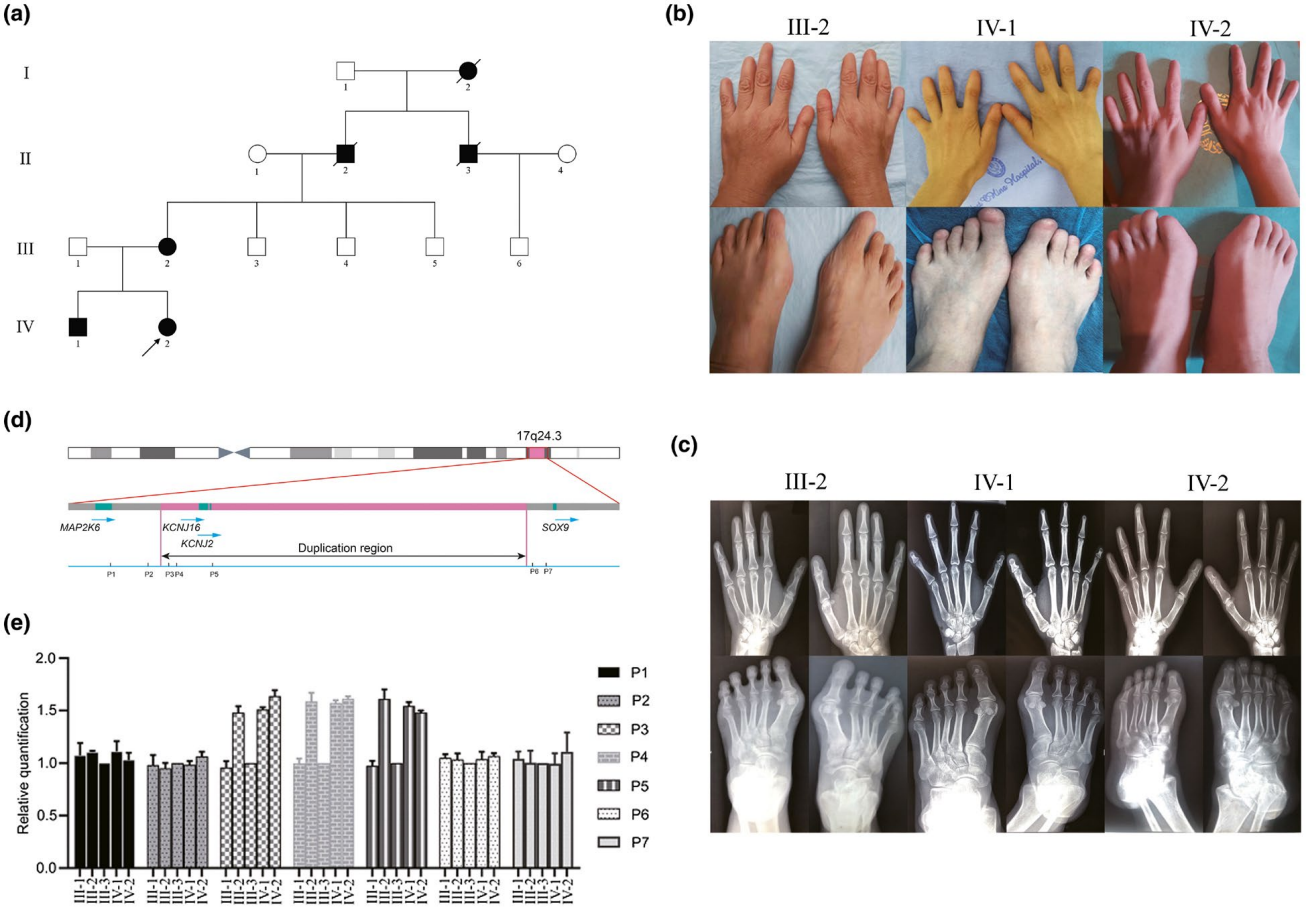
Brachydactyly‐anonychia caused by 17q24.3 duplication. (a) The pedigree structure of the family. Squares represent male pedigree members, circles represent female pedigree members, solid symbols represent members with brachydactyly‐anonychia, oblique lines represent dead members, and open symbols represent unaffected members; the proband is indicated by black arrows. (b) The clinical phenotype of the affected individuals in this family. The patients represent shortened digits II‐V with no fingernails. (c) The radiographs of the hands and feet of the patients. The absence of middle phalanges of finger/toes II‐V was observed in all patients. (d) The schematic illustration of 17q24.3 duplication. The red bars indicate the duplication region, the green bars indicate the positions of *MAP2K6* (NM_002758.4), *KCNJ16* (NM_170741.4), *KCNJ2* (NM_000891.3), and *SOX9* (NM_000346.4), and P1–P7 indicates the localization of qPCR amplicons on chromosome 17. (e) The duplication detected by chromosome microarray was confirmed by qPCR. The affected individuals showed the increased relative copy numbers for amplicons in the duplication region (P3‐P5) compared to the unaffected relatives. All the people in the family showed similar relative copy numbers for amplicons out of the duplication (P1, P2, P6, and P7). qPCR, quantitative real‐time PCR

The proband was a 25‐year‐old woman who showed short fingers and toes as well as missing nails. After genetic counseling, we got the information that there were three affected individuals in this family with no consanguineous marriage. All patients in this family presented with brachydactyly‐anonychia, and had the normal stature as well as intellectual development and no other skeletal abnormality (Figure 1a). Among them, three members (I‐2, II‐2, and II‐3) were dead and said to have ‘‘shortened fingers/toes and absent nails’’ by their family members; and the three identified patients (III‐2, IV‐1, and IV‐2) exhibited a distinct clinical phenotype: the shortening and missing middle flexion creases of figures II‐V on hands as well as feet, and absent nails in all digits (Figure 1b). Furthermore, X‐ray examination was carried out on the three affected individuals. They were characterized by missing of middle phalanges in figure II‐V of the upper and lower extremities (Figure 1c).

### 3.2 Molecular genetic studies

The findings in our study indicated that the pedigree suffered from genetic BD. However, through whole‐exome sequencing (WES), we detected no pathogenic mutations in genes conferred to bone development. Because some CNVs, including deletions and duplications, cannot be readily detected by WES, we also employed comparative genomic hybridization (CGH) for CNV analysis on this family. Remarkably, a 1.9 Mb duplication in chromosome 17q24.3 (Chr17: 67892996–69792434) was identified only in the three affected individuals, which is involved in two coding regions of *KCNJ2* and *KCNJ16*, and the 5′ regulatory region of *SOX9* (Figure 1d). We next confirmed this duplication by qPCR (Figure 1e). To further verify the potential pathogenicity of this CNV, we conducted qPCR analysis to screen for this duplicated region in 500 normal controls and no one carried this variation. Strikingly, Kurth et al. (2009) have detected four overlapping duplications in a ~2 Mb interval on chromosome 17q24.3 in seven affected individuals from four unrelated families with brachydactyly‐anonychia, and the mechanism was further unveiled by Martin Franke et al. (2016). Therefore, we suggested that the duplication in 17q24.3 was associated with brachydactyly‐anonychia in this family.

## 4 DISCUSSION

Here, we reported three patients with brachydactyly‐anonychia from a large Chinese family. By CNV analysis, we identified a pathogenic duplication in 17q24.3 in all affected family members.

Most of the known BD are associated with oligogenic variations in the coding regions or the splice sites.^3–8^ However, both we and Ingo Kurth et al. (Franke et al., 2016) discovered that the duplications in 17q24.3 influencing the coding sequences of *KCNJ2* and *KCNJ16* and the noncoding elements 5′ of *SOX9* could cause solitary brachydactyly‐anonychia. Intriguingly, Franke et al. (2016) further uncovered the pathogenesis of this kind of brachydactyly‐anonychia, suggesting that a new chromatin domain (neo‐TAD) was formed by 17q24.3 duplications which incorporated the next flanking gene, *Kcnj2*, and resulted in ectopic contacts of *Kcnj2* with the *Sox9* regulatory region, subsequent misexpression of *Kcnj2*, and a limb malformation phenotype. To date, the pathogenicity of this CNV was not approved by public database because of the lack of more evidences. Fortunately, our three patients provide strong evidence that 17q24.3 duplications related to regulatory elements of *SOX9* as well as *KCNJ2* coding region are a genetic cause of brachydactyly‐anonychia.

Actually, genes associated with development are thought to have complex expression regulation patterns, some of which are involved in cis‐acting regulatory control elements located in about 1.5 Mb in either direction of the transcription unit. Any change destroying the regulatory elements may cause the gaining or missing of enhancer function, thus dysregulating the related gene expression and further leading to disorders. Zone of polarizing activity (ZPA) and apical ectodermal ridge (AER) play the pivotal role in limb formation. Strikingly, mutations in their coding regions usually cause additional phenotypes except limb malformations; however, variants in enhancers only lead to limb phenotypes (Akiyama, Chaboissier, Martin, Schedl, & de Crombrugghe, 2002). Another research observed the consistent results that changing the mouse *Prx1* limb enhancer only affect the limb morphology, but no other phenotypes (Cretekos et al., 2008). Dominant mutations in *KCNJ2* have been suggested to associate with Andersen‐Tawil syndrome (MIM170390), a channelopathy characterized by periodic paralysis, ventricular arrhythmias, and dysmorphic facial or skeletal features. However, here, 17q24.3 duplications disrupt the boundary between SOX9 TAD and KCNJ TAD, leading to overexpression of *Kcnj2* in the digit anlagen and limb buds, and further brachydactyly‐anonychia but no any phenotypes related to Andersen‐Tawil syndrome (Franke et al., 2016). Collectively, it is conceivable that alterations in expression regulatory region are associated with the gene neofunctionalization.

In conclusion, our clinical data described a BD characterized by complete absence of middle phalanges of finger/toes II‐V in combination with anonychia. We further identified that 17q24.3 duplication was the causative variation of brachydactyly‐anonychia, which could be used for gene diagnosis for this disease in the future.

## CONFLICT OF INTEREST

The authors declare no conflict of interests.

## AUTHOR’S CONTRIBUTION

Hongqian Liu collected data and conducted the clinical evaluations. Xueguang Zhang performed qPCR. Mohan Liu performed genetic analysis and wrote the first manuscript. Ying Shen supervised the study and revised the manuscript. All authors revised and approved the article.

## Supporting information


**Table S1**

## Data Availability

The data that support the findings of this study are available on request from the corresponding author. The data are not publicly available due to privacy or ethical restrictions.
