# Supplementary material for: A 17q24.3 duplication identified in a large Chinese family with brachydactyly‐anonychia
Source: Mol Genet Genomic Med. 2020 Jun 25;8(9):e1392. doi: 10.1002/mgg3.1392 (PMC7507485; doi:10.1002/mgg3.1392)
Supplement: Supplementary file 1 — Table S1 [file MGG3-8-e1392-s001.docx]

| Primer | Sequence 5’ → 3’ | Amplicon position on  chr. 17 [bp] |
| --- | --- | --- |
| P1-F  P1-R | TTTATCACTGGGTTTCTGGC  TTCTTGTGGCATTTGGCTA | 67604967-67605321 |
| P2-F  P2-R | CCAAAGAGACTCAGGTCATCC  CTCGTGGTCCAGAGACTAAAT | 67890121-67890245 |
| P3-F  P3-R | TTTTTCCCTGCTTTTGCG  GTTGCCCTGCGGCTATCT | 67900376-67900545 |
| P4-F  P4-R | TATTGCCATCACAGTTCCTC  ATAAGCGTCCCTTTTTCTCC | 67915624-67915870 |
| P5-F  P5-R | ATTCCACTGCTACTTGCCC  CTAACCATCCCCTCACCTC | 68130074-68130342 |
| P6-F  P6-R | AAGGTAGAAGAGATGAGAGAGTAGG  GGCAACAGGAGTAGCAGGAAA | 69794780-69795053 |
| P7-F  P7-R | GACCCTTGTCTATTCTCTTTGC  TGTCTAATCTGCTTATCCTGCT | 69840921-69841348 |

**Table S1**. **The information regarding the PCR primers used in the present study**
